# On Nux Vomica in Impotence and Spermatorrhœa

**Published:** 1850-01

**Authors:** 


### MATERIA MEDICA AND THERAPEUTICS.

*On Nux Vomica in Impotence and Spermatorrhœa.* By M. Duclos. —Incomplete impotence is of far more common occurrence than would be supposed, until many patients have been questioned respecting it. Erections are almost always possible, especially in the morning; but they are soft, incomplete, and insufficient, a certain amount of tension only continuing, and that for a short time. This state may be met with in men even of the strongest make and most robust constitution, in whom the vascular and muscular systems have attained their highest development. In others, in whom these systems and the nervous system are ill-developed, the generative functions are properly exercised; so that the general physical force is no criterion of the special force of these organs. This imperfect condition is as often found in those who have been excessively continent, as in those who have abused the sexual organs; and it is observed just as often in persons whose nervous system is easily excitable, as in those in whom its lesser irritability allows of a predominance of the muscular and vascular systems. Self-pollution may occur either by night or by day, the discharge being either a true or a pseudo-spermatorrhœa.

Accident first led the author to the employment of nux vomica in this class of affections; and he has since observed several cases in which its efficacy has proved very great. He divides 75 grains of the alcoholic extract into 100 pills. During five days he gives 1 every night; then for other 5 days, 1 morning, 2 night; for other 5 days, 2 night and morning; and for other 5 days, 2 morning and 3 at night; and so on until 4 are taken night and morning. He has never found any harm result, although some patients have taken 14 pills per diem. In many cases the stomach is rapidly improved by the medicine, the lost appetite returning. The following liniment rubbed into the loins and on the inside of the thighs is a valuable though not an essential auxiliary:— R. Træ. Nuc. Vom., Træ. Arnicæ vel Melissæ, aa, 60 p. Tr. Lyttæ 15 p. The regimen should be tonic; and the increased appetite demands a larger supply of food. A very moderate use of coitus is advisable.—*Brit. and For. Medico-Chirug. Rev., from Bull. de Therap.*

